# Global characterization of Artemisia annua glandular trichome transcriptome using 454 pyrosequencing

**DOI:** 10.1186/1471-2164-10-465

**Published:** 2009-10-09

**Authors:** Wei Wang, Yejun Wang, Qing Zhang, Yan Qi, Dianjing Guo

**Affiliations:** 1 Department of Biology and the State Key Lab for Agrobiotechnology, The Chinese University of Hong Kong, Shatin, Hong Kong SAR, PR China

## Abstract

**Background:**

Glandular trichomes produce a wide variety of commercially important secondary metabolites in many plant species. The most prominent anti-malarial drug artemisinin, a sesquiterpene lactone, is produced in glandular trichomes of *Artemisia annua*. However, only limited genomic information is currently available in this non-model plant species.

**Results:**

We present a global characterization of *A. annua *glandular trichome transcriptome using 454 pyrosequencing. Sequencing runs using two normalized cDNA collections from glandular trichomes yielded 406,044 expressed sequence tags (average length = 210 nucleotides), which assembled into 42,678 contigs and 147,699 singletons. Performing a second sequencing run only increased the number of genes identified by ~30%, indicating that massively parallel pyrosequencing provides deep coverage of the *A. annua *trichome transcriptome. By BLAST search against the NCBI non-redundant protein database, putative functions were assigned to over 28,573 unigenes, including previously undescribed enzymes likely involved in sesquiterpene biosynthesis. Comparison with ESTs derived from trichome collections of other plant species revealed expressed genes in common functional categories across different plant species. RT-PCR analysis confirmed the expression of selected unigenes and novel transcripts in *A. annua *glandular trichomes.

**Conclusion:**

The presence of contigs corresponding to enzymes for terpenoids and flavonoids biosynthesis suggests important metabolic activity in *A. annua *glandular trichomes. Our comprehensive survey of genes expressed in glandular trichome will facilitate new gene discovery and shed light on the regulatory mechanism of artemisinin metabolism and trichome function in *A. annua*.

## Background

Secreting glandular trichomes (GTs) are a major site for biosynthesis and accumulation of a wide range of plant natural products. These plant natural products often function to protect the plants against insect predation [1,2], and contribute to the flavour and aroma of plants. Many of the natural products also have pharmacological effects, such as the analgesic drug morphine, the anticancer compound taxol, and the antimalarial drug artemisinin. Artemisinin, a sesquiterpene lactone, is currently recognized as one of the most prominent anti-malarial treatment [3]. A complete understanding of the artemisinin biosynthetic pathway and its regulatory mechanism holds the key to efficient metabolic engineering for increased artemisinin yield. In the past decades, research efforts have been dedicated to identification of enzymes and intermediate compounds leading to artemisinin production. Many genes encoding enzymes participate in the pathway have been cloned and functionally characterized [4-10]. However, little is known about the regulatory aspects of sesquiterpene metabolism. This is partly due to the fact that *A. annua *is a non-model plant with limited genomic information available, and sequencing of limited number of randomly selected cDNA clones often have insufficient coverage of less abundant transcripts, including important regulatory transcription factors (TFs). In addition, genes uniquely or preferentially expressed in trichomes may be under-represented in non-tissue-targeted EST sequencing projects. A comprehensive survey of genes expressed in glandular trichome will facilitate new gene discovery and contribute significantly to elucidating the terpenoid pathway regulation and trichome function in *A. annua*.

Whole genome or transcriptome sequencing enables functional genomic studies based on global gene expression. The newly developed high throughput pyrosequencing technology allows rapid production of sequence data with dramatically reduced time, labor, and cost [11-15]. So far, most applications of pyrosequencing have involved analysis of genomic DNA [16]. Published reports on 454 pyrosequencing of transcriptomes have been mostly restricted to model species with genomic or comprehensive Sanger EST data available [11,17-19]. Previous studies [11,19] using genome or Sanger EST sequences for mapping and annotation of 454 ESTs were not able to accomplish *de novo *assembly of their 454 ESTs. We here present the global transcriptome characterization of *A. annua *glandular trichome, the so called biofactory for the production of artemisinin and other plant secondary metabolites. We assigned putative function to 28,573 unigenes, including previously undescribed enzymes likely involved in sesquiterpene biosynthesis. We verified the expression of 32 selected unigenes and novel transcripts in glandular trichomes using semi-quantitative RT-PCR. These 454 ESTs were linked to metabolic process specific in glandular trichomes and form the basis for further investigation.

## Results

### Sequencing and assembly of 454 pyrosequencing ESTs

Totally 406,044 ESTs (minimal size > 50 bp) averaging 210 bp were generated from two consecutive pyrosequencing runs. Cleaning (removal of primer, polyA tail, etc.) of the raw sequences resulted in a total of 386,881 high quality reads with an average length of 205 nucleotides totalling 85 Mb. After clustering and assembly using TGICL CAP3 clustering tools [20,21], these reads were assembled into 42,678 contigs and 147,699 singletons. The average length for contigs and singletons are 334 bp and 191 bp respectively. The contigs and singletons are collectively referred to as unigenes. The length distribution of unigenes and their component reads are summarized in Table 1 and Table 2.

**Table 1:** Length distribution of assembled contigs and singletons

**Nucleotides length (bp)**	**Contigs**	**Singletons**
50-99	276	22,730
100-199	2,534	41,936
200-299	19,220	82,169
300-399	11,568	863
400-499	4,940	1
500-599	1,991	0
600-699	980	0
700-799	529	0
800-899	296	0
900-999	142	0
1,000-1,499	173	0
1,500-1,999	22	0
> 2,000	7	0

Total	42,678	147,699
Maximum length	2,366 bp	411 bp
Average length	334 bp	191 bp

**Table 2:** Summary of component reads per assembly

**Number of reads**	**Number of contigs**
2 to 10	39,112
11 to 20	2,142
21-30	585
31-40	289
41-50	164
51-100	250
101-150	63
151-200	27
> 200	46

### Pyrosequencing provides deep coverage of the *A. annua *trichome transcriptome

The contigs were searched against the NCBI non-redundant (NR) protein database using the blastx algorithm. Among the 190,377 contigs and singletons, 29,577(15.5%) had at least one significant alignment to existing gene model in blastx searches (E-value cutoff, e-^10^) (see Additional file 1). A majority (84.5%) of the pyrosequencing assemblies did not match any known sequences in the existing database and thus likely represent novel (E-value cutoff, pts sion of 17 transcripts identified in this study. Performing a second sequencing run increased the number of genes identified by approximately 30% (Table 3), suggesting that two pyrosequencing runs detect a substantial fraction of genes expressed in glandular trichomes and provide deep coverage of the *A. annua *trichome transcriptome.

**Table 3:** Summary of blast hits from two pyrosequencing runs

**Pyrosequencing run**	**NCBI database unique hits**
1^st ^run	266,976
2^nd ^run	289,467
Total	357, 843

### Characterization and GO annotation of novel transcripts

**Figure 1:**
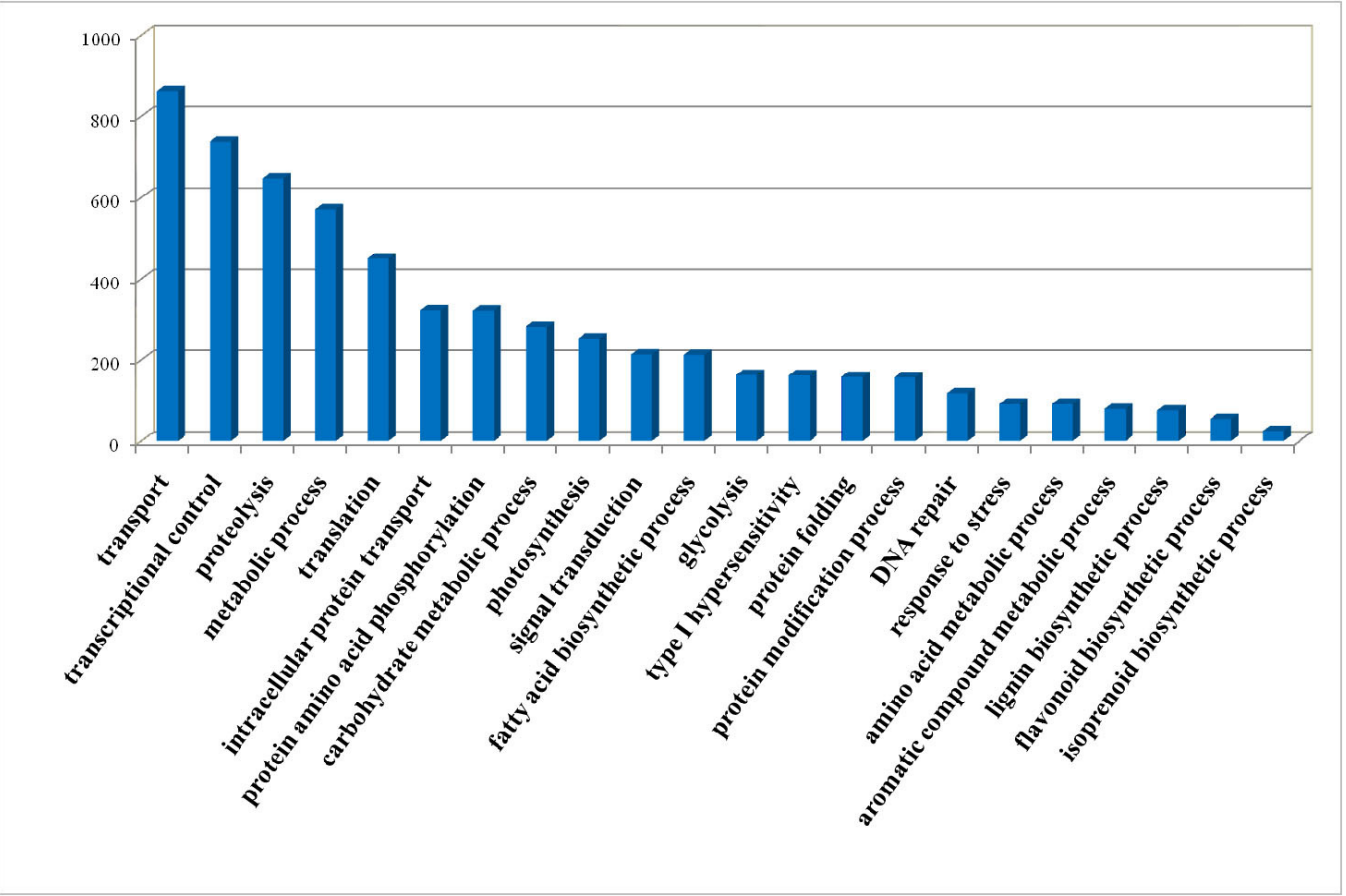
**Top-ranked GO categories (molecular function) of assembled pyrosequencing ESTs**.

The 357,843 sequences that had matches with protein sequences in the NCBI protein database  could be condensed into 29,577 clusters based on their top protein hits. Each 454 contig was assigned a putative gene description and a GO classification based on the 'best hit' blastx search (bitscore > 45, e-value < 1-10), using the 'inferred from sequence similarity' (ISS) level of evidence [22]. The unigenes were classified into three major functional categories: biological process, molecular function, and cellular component, according to the standard Gene Ontology terms (GO; ). The assigned functionality of genes covers a broad range of GO categories. The top 20 most highly represented GO categories are illustrated in Figure 1. Under the category of biological process, transport, transcriptional control, and metabolic process were among the most highly represented categories, indicating the important metabolic activities in *A. annua *glandular trichomes. Other categories include photosynthesis, secondary metabolism (lignin, flavonoid, and isoprenoid biosynthesis process) and primary metabolism (fatty acid, glycolysis, carbohydrateprocess etc.).

### Comparison of 454 sequence contigs to trichome ESTs from other plant species

TrichOME  is a publicly available database of genes and metabolites expressed in plant trichomes. It currently contains 37, 017 conventional ESTs derived from 8 plant species, including *Medicago sativa*, *Humulus lupulus*, *Mentha × piperita*, *Nicotiana benthamiana*, *Ocimum basilicum*, *Solanum habrochaites*, *Solanum lycopersicum *and *Solanum pennellii*. A tblastx search against TrichomeDB showed that only 17,372(9%) of our 454 contigs had best blast hits (e-value < 1e-10) to 8,095 EST clusters with unique descriptions. Thus 454 sequencing has revealed many transcripts not previously detected in *A. annua*. ESTs homologous to photosynthesis-related proteins (chlorophyll a/b binding protein, ribulose bisphosphate carboxylase small subunit) are among the top 10 most highly expressed transcripts. The top ranked common molecular function of ESTs identified from all 9 plant species are listed in Table 4. Regulation of metabolic process, metabolic process, oxidation reduction, and transport categories has the highest number of contigs. Trichomes are known to be active in photosynthesis, as well as for their roles in storage and secretion of toxic compounds e.g. heavy metals [23,24], which requires the function of transporters. In our assembled pyrosequencing EST collections, we identified a large number of contigs homologous to ABC transporter, which is one of the most important families of membrane transport proteins that may play critical roles in the transmembrane transport of secondary metabolites in plants. The large amount of transporters can be linked to the secretion and transport function of glandular trichomes.

**Table 4:** Shared common GO terms (biological process) in all trichome EST databases

**GO ID**	**GO term**	**No. of unigenes**
GO:0006464	Positive regulation of protein metabolic process	147
GO:0006730	Metabolic process	36
GO:0008152	Positive regulation of metabolic process	28
GO:0055114	Oxidation reduction	21
GO:0006006	Glucose metabolic process	21
GO:0006334	Nucleosome assembly	16
GO:0006412	Positive regulation of biosynthetic process	14
GO:0006096	Positive regulation of glycolysis	5
GO:0006810	Transport	5
GO:0006869	Positive regulation of lipid transport	2

### Representation of genes related to secondary metabolism

**Figure 2:**
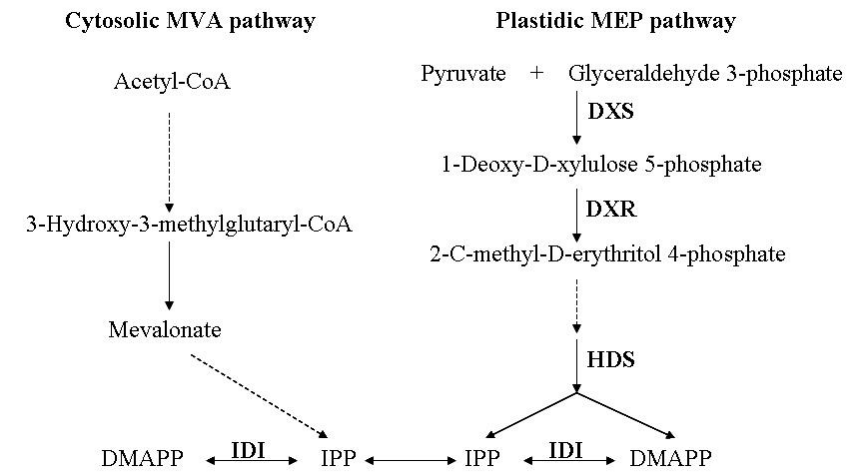
**Simplified graphical representation of terpenoid biosynthetic pathway in *A. annua***. DXR: deoxy-D-xylulose 5-phosphate synthase; DXP: 1-deoxy-Dxylulose-5-phosphate reductoisomerase; HDS: 1-hydroxy-2-methyl-2-(E)-butenyl 4-diphosphate synthase; IDI: isopentenyl diphosphate/dimethylallyl diphosphate isomerase. DMAPP: Dimethylallyl Diphosphate. IPP: isopentenyl diphosphate.

Numerous sesquiterpene and monoterpene compounds have been identified in *A. annua *leaves, stems [5,25,26] and isolated glandular trichomes [27]. The genes corresponding to enzymes involved in the biosynthesis of major sesquiterpenes have been cloned and characterized [5,25-33]. To investigate the trichome function in secondary metabolism, the annotated unigenes were searched for enzymes participate in terpenoids biosynthesis. As shown in Additional file 2, unigenes corresponding to all the known enzymes in the terpenoids MEP and MVA pathway were identified. In higher plants, terpenoids precursor isopentenyl diphosphate (IPP) can be produced from both MVA and MEP routes, which is then converted to its isomer DMAPP (Figure 2) [34]. The cytosolic MVA terpenoids pathway, which starts from acetyl-CoA and proceeds through the intermediate mevalonate (MVA), provides the precursors for sterols and ubiquinone [35]. The plastidial MEP pathway, which involves a condensation of pyruvate and glyceraldehyde-3-phosphate, is used for the synthesis of isoprene, carotenoids, abscisic acid, and the side chains of chlorophylls and plastoquinone [36-39]. Although the subcellular compartmentation allows both pathways to operate independently, there is ample evidence that cross-talk exist between these two pathways [40,41].

Unigenes encoding the MEP and MVA pathway enzymes and all the sesquiterpene artemisinin pathway enzymes were present in our pyrosequencing collection. It is noteworthy that although the sequences were derived from normalized cDNA collections, unigenes corresponding to MEP pathway enzymes were two fold more abundant as compared with MVA pathway transcripts. This likely suggests that the MEP pathway may serve as a major route for DMAPP/IPP production in the *A. annua *trichomes. The MEP pathway has previously been shown to provide precursors for both mono-and sesqui-terpene biosynthesis in snapdragon flowers [42]. In a recent report on hops, the ESTs encoding MEP pathway enzymes are also found more abundant than those of MVA pathways [43].

**Figure 3:**
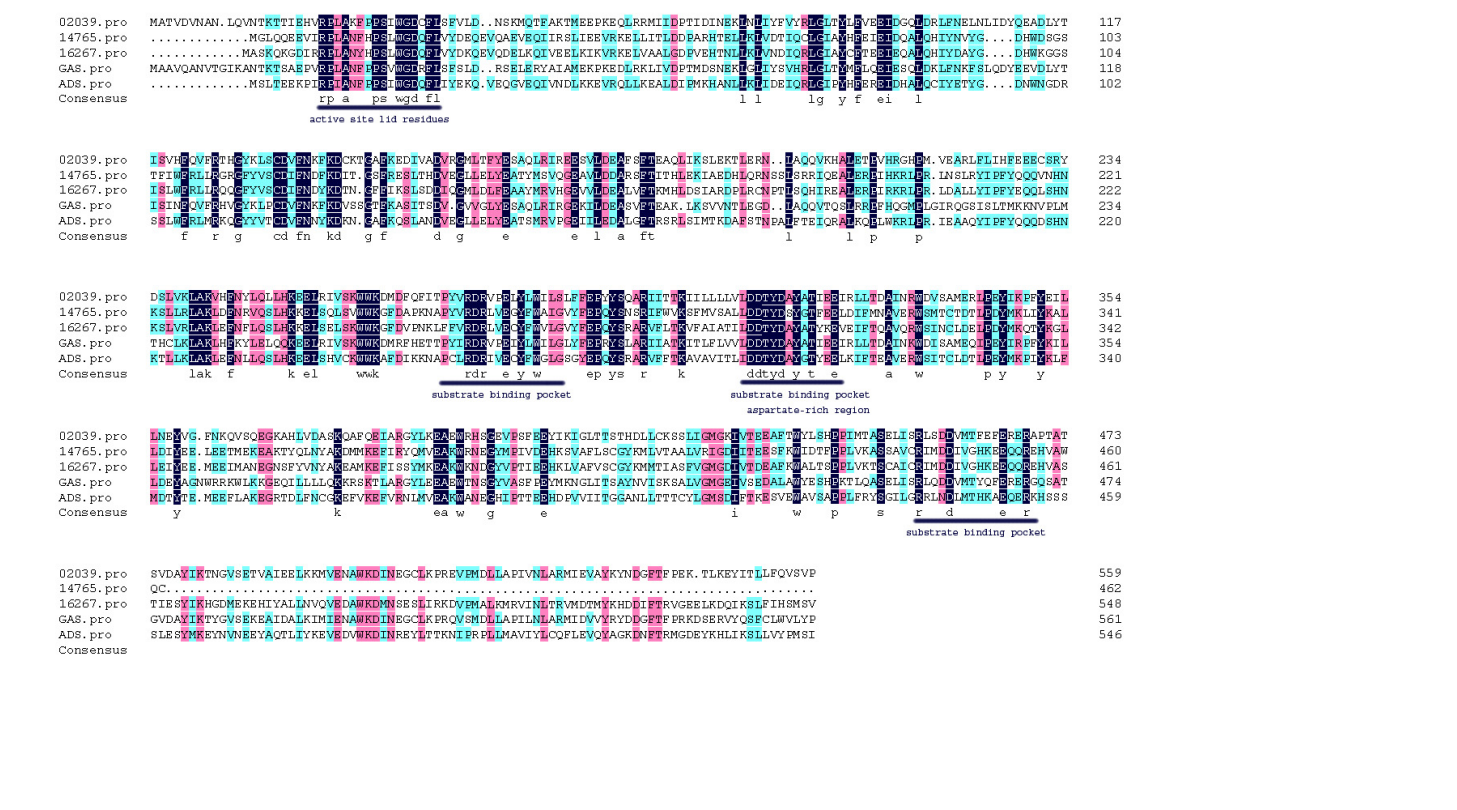
**Alignment of putative sesquiterpene synthases with other homologs from A. annua **(accession no. DQ447636 and AY006482). Identical amino acids are highlighted. The functional motifs are underlined.

Except for those well characterized terpenoid pathway genes, other unigenes annotated as sesquiterpene synthase and monoterpene synthase were identified. Three unigenes (Contig02039, Contig16267, Contig14765) annotated as sesquiterpine synthases were selected for RACE PCR to retrieve the full length cDNAs. Sequence analysis indicated that the conserved sesquiterpene synthase functional domain exists in all three genes (Figure 3). Further functional characterization of these enzymes will be reported elsewhere.

Furthermore, large amount of unigenes annotated as phenylpropanoids and flavanoids pathway enzymes were present in the assemebled pyrosequencing EST collection (ss Additional file 3), indicating the metabolic function of glandular trichomes in *A. annua *secondary metabolism.

### RT-PCR validation

**Figure 4:**
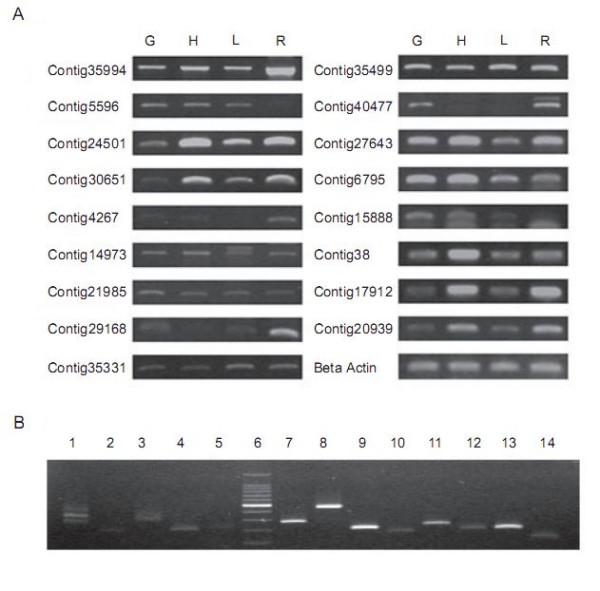
**Semi-quantitative RT-PCR analysis of selected unigenes and novel transcripts**. A. Expression of selected contigs in different tissue types. G: Glandular trichome, N: Non-glandular tirhcome, L: Leaf, R: Root. bHLH family proteins: Contig35994 and Contig5596; WD family proteins: Contig24501 and Contig30651; Myb family proteins: Contig14973, Contig21985, Contig29168, Contig35331, Contig35499, and Contig40477; Terpene synthases: Contig27643; amorpha-4,11-diene synthase [Artemisia annua] ABM88787: Contig 6795; sesquiterpene cyclase [Artemisia annua] AAG24640: Contig15888 (3R)- linalool synthase [Artemisia annua] AAF13356; WRKY Proteins: Contig38, Contig17912, Contig20939. B. Expression of novel transcripts and singletons in GT. Lane 1-5: singletons S122859, S078690, S091943, S154166, and S174533; Lane 6: DNA marker; Lane 7-14: novel transcripts C3719, C13021, C15708, C1441, C20920, C29103, C445, and C14916.

A set of 17 contigs were selected for semi-quantitative RT-PCR analysis to confirm their expression (Figure 4A). The selected contigs encode enzymes involved in artemisinin biosynthesis, and putativetranscription factors. PCR experiments were conducted on four pools of cDNAs derived from (1) glandular trichomes, (2) non-glandular trichomes (3) leaves, and (4) roots. The results demonstrate that all of the novel transcripts detected among the 454-ESTs are indeed expressed in glandular trichomes, including those with low expression levels. This suggests that deep pyrosequencing is effective in revealing the expression of many rare transcripts, e.g. transcription factors. Most of the tested contigs were also expressed in leaf and non glandular trichome cDNA pools, except for one contig40477, which was only expressed in glandular trichomes and roots. Interestingly, three contigs likely encode enzymes needed in sesquiterpene biosynthesis were also strongly expressed in non-glandular type of trichomes. This raises the question as to whether glandular trichome is the sole site for the biosynthesis of artemisinin and other sesquiterpenes in *A. annua*.

RT-PCR was also used to confirm the expression of novel transcripts and singletons. A set of 18 novel transcripts and singletons was randomly selected to test if they are indeed expressed in GT (Figure 4B). Of the 20 primer pairs, 13 produced RT-PCR products that were of the correct size and whose sequence matched the sequences from which the primers were designed. Based on these results, we conclude that many of the novel transcripts and singletons detected among the 454-ESTs are not due to the sequencing artifacts. This result provides further evidence for the value of tissue specific 454 sequencing for gene discovery.

## Discussion

As the sole plant source for artemisinin production, the *A. annua *has been studied extensively for the past decades. Like most other non-model plant species, it has lacked genetic and genomic resources necessary for mechanistic study. Although a precise estimate of transcriptome coverage is unattainable without full genomic sequence, we appear to have recovered a significantly portion of the *A. annua *glandular trichome transcriptome. Novel transcripts detected highlights the hypothesis-expanding aspects of 454 deep pyrosequencing approach, which potentially facilitate the understanding of glandular trichome metabolic function. The assembled sequence data also provided a rich source of information for further investigation.

Two consecutive pyrosequencing runs identified a large number of genes expressed in glandular trichomes. In data analysis, approximately 85% of the pyrosequencing assemblies did not align to any ESTs available in GenBank. This high proportion could reflect the specialized cell type that was sampled or perhaps the greater complexity of the *A. annua *genome. Because our priority goal in this study is gene discovery, we therefore chose normalized cDNA population to reduce oversampling of abundant transcripts and to maximize coverage of less abundant transcripts present in the sample. The average contig length was fairly short (~334 bp), and only 62% of the sequence reads assembled into contigs, leaving 147,699 singletons.

Genes involved in plant secondary metabolism have frequently been identified by EST approach [44]. The lower cost and greater sequence coverage offered by pyrosequencing makes it possible to identify more candidate genes involved in plant natural product biosynthetic pathways, esp. those with low abundance and often missed by conventional EST projects. For non-model species with little or no genomic data available, such as *A. annua*, pyrosequencing offers rapid characterization of a large portion of the transcriptome and therefore provides a comprehensive tool for gene discovery. However, one limitation of pyrosequencing is that one must rely on RACE PCR in order to obtain full-length sequence data for a given gene of interest.

Comparison between our glandular trichome 454 ESTs with conventional ESTs generated from trichomes of other plant species revealed likely common function in non-glandular and glandular trichomes. In addition, some unigenes corresponding to enzymes in sesquiterpene biosynthesis were found to be highly expressed in both trichome types in our RT-PCR analysis. Although it has been suggested that glandular trichomes are the site for synthesis and accumulation of plant secondary metabolites, it will be interesting to further investigate the different functional roles of non-glandular trichomes in artemisinin biosynthesis.

## Conclusion

In conclusion, we describe the global analysis of glandular trichome in *A. annua *using massively parallel pyrosequencing. Mining the pyrosequencing ESTs resulted in the identification of many contigs likely involved in terpenoid biosynthesis and trichome function. Functional characterizations of selected genes are being carried out. These pyrosequencing data form the basis for further characterization of the molecular mechanism of glandular trichome function in *A. annua*. The results also highlight the value of using tissue-specific high throughput pyrosequencing technology for gene discovery in non-model plants. Access to all EST contigs obtained in this study is facilitated through a file available in the supplemental data (see Additional file 4).

## Methods

### Plant Materials

*A. annua *seeds were purchased from Youyang, Sichuang province of China. Seeds were sown into commercial potting mixture for germination. The germinated plantlets were grown under natural light conditions in the greenhouse located at The Chinese University of Hong Kong. Flower buds were collected for trichome isolation before flowering.

### Isolation of glandular trichomes

**Figure 5:**
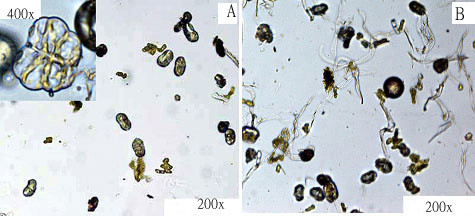
**Isolation of glandular trichomes**. A: Extracted glandular trichomes B: Crude extracts containing both glandular and non-glandular trichomes.

Trichome cells were gently abraded from the surface of flower buds using glass beads and a commercial cell disrupter (BioSpec Products). The isolated secretary cells were separated from other cells and tissue fragments in the mixture by sequentially passing through a 40 μm and a 30 μm nylon sieves. Glandular cells were finally collected in 30 μm meshes with minimum contamination of non-glandular trichomes (Figure 5).

### RNA extraction, cDNA synthesis and normalization

Total RNA was extracted from glandular trichomes isolated from 30 g flower buds following the standard protocol of RNeasy Plant Mini Kit (Qiagen). cDNA was synthesized using the BD SMARTM PCR cDNA Synthesis Kit (Clontech). First-strand cDNA synthesis was performed with oligo(dT) primer as described in the provided protocol using 500 ng total RNA. Double-strand cDNA was prepared from 2 μl of the first-strand reaction by PCR with provided primers in a 100 μL reaction. cDNA was purified using Qiagen QIAquick PCR purification spin columns. Normalization was performed using TRIMMER cDNA normalization kit (EVR_GEN) to decrease the prevalence of abundant transcripts before sequencing. Approximately 1 μg of normalized double stranded cDNA was used for 454 pyrosequencing.

### 454 pyrosequencing, data pre-process and assembly

Approximately 1 μg of the adaptor-ligated cDNA population was sheared by nebulization and DNA sequencing was performed following protocols for the Genome Sequencer GS FLX System (Roche Diagnostic). Reads generated by the FLX sequencer were trimmed of low quality, low complexity [poly(A)] and adaptor sequences using the SeqClean software . The cleaned sequences were subject to CAP3 program [20] for clustering and assembly using default parameters.

### Gene annotation using GO terms

After assembly, the resulting contigs and singlets were aligned with NCBI non-redundant protein database using blast2go software with a cut-off e-value of 1e-10. The GI accessions of best hits were retrieved, and the GO accessions were mapped to GO terms according to molecular function, biological process and cellular component ontologies http://www.geneontology.org/.

### Semi-quantitative RT-PCR analysis

To verify the presence of pyrosequencing ESTs in glandular trichomes, we totally selected 35 unigenes and novel transcripts for RT-PCR analysis. Total RNA were extracted from glandular trichomes, non-glandular hairy trichomes, leaves and hairy roots respectively. The first-strand cDNA was synthesized from 10 μL (about 1 μg) total RNA using SuperScript™ II Reverse Transcriptase (Invitrogen) with Oligo(dT)12-18 Primer. PCR was performed using 0.5 to 2 μL of the cDNA in a total of 50 μL reaction volume. The PCR conditions were 2 min at 95°C, 30 s at 95°C, 30 s at 47-56°C, 1 min at 72°C for 30 cycles, followed by 5 min at 72°C. These conditions were chosen because none of the samples analyzed reached a plateau at the end of the amplification (i.e. they were at the exponential phase of the amplification). Actin was used as a loading control, and loading was estimated by staining the gel with ethidium bromide. Expression analysis of each gene was confirmed in at least 2 independent RT-reactions using forward and reverse primers.

## List of abbreviations used

GTs: glandular trichomes; TFs: transcription factors; NR: non-redundant; EST: expressed sequence tag; ABC transporter: ATP-binding cassette transponer; *A. annua*: *Artemisia annua*; GO: gene ontology; ISS: inferred from sequence similarity; MEP pathway: 2-C-methyl-d-erythritol 4-phosphate pathway; MVA pathway: mevalonic acid pathway; IPP: isopentenyl diphosphate; DMAPP: Dimethylallyl pyrophosphate.

## Authors' contributions

WW carried out the trichome isolation, RT-PCR, and participated in the sequence analysis and drafted the manuscript. YW and QZ carried out EST assembly, data annotation and bioinformatics analysis. YQ participated in the trichome isolation and sequence analysis. Dianjing Guo conceived of the study and and participated in its design and coordination. All authors read and approved the final manuscript.

## Supplementary Material

Additional file 1 **Contigs with at least one significant alignment to existing gene model**. The data represent all the assembled contigs with at least one significant alignment to the existing gene model according to BlastX search. Click here for file

Additional file 2 **Unigenes encoding putative enzymes in terpenoids metabolism**. The data represent all the unigenes encoding putative enzymes in terpenoids metabolism. Click here for file

Additional file 3 **Unigenes annotated as phenylpropanoids and flavanoids pathway enzymes presented in assembled pyrosequencing EST collection**. The data represent all the unigenes annotated as phenylpropanoids and flavanoids pathway enzymes. Click here for file

Additional file 4 **Assembled pyrosequencing ESTs**. The data represent all the assembled pyrosequencing ESTs. Click here for file
